# A Revolution in Gas Lighting

**Published:** 1898-06-11

**Authors:** 


### A REVOLUTION IN GAS LIGHTING.

The greatly increased use of electric lighting for private houses and for the purposes of street illumination of late years has had one indirect but very important result. Those interested in the future of gas as a lighting medium have been compelled to face the fact that if the old system were to hold its own at all against the new, vast improvement must speedily take place. The ancient and long familiar "fishtail," or flat-flame burner, in all its native hideousness and its associations of heat and smell, could no longer be tolerated, and recent years—especially the last one or two—have seen great developments in incandescent gas lighting. The latest of these seems to give promise that electric lighting is not yet to carry all before it. The name of Dr. Carl Auer von Welsbach is now well known in connection with his Incandescent Gas Light system, an exhibition of which attracted much attention in 1887. The recent exhibition held at the Niagara Hall, Westminster, showed all who visited it how tremendous a step forward has been taken of late in the matter of gas lighting. The new burner, which is only just being placed on the market, was the centre of interest. It is a special form of Bunsen burner, by which it is claimed that, at an ordinary pressure of from six-tenths to eight-tenths with the ordinary gas, without any other mechanical means, a far more complete mixture of gas and air is obtained, the result of the larger admixture of air being a flame greatly superior to any hitherto produced. Its light-giving power is, with normal pressure, from 25 to 30 candle power per cubic foot of gas consumed, with all sizes of burners. Increase of pressure, instead of being a difficulty, has been found by experiment only to make the flame hotter and the light more brilliant. One of its chief advantages is that no chimney is required, a very awkward appendage being thus cleared out of the way, and the charming appearance presented by the globes on view at the exhibition demonstrated that for decorative purposes a result equal in effect to electric lighting has been achieved in this far more economical form of illumination. For institutional use the improved incandescent gas lighting must prove very valuable on account of the greatly increased power of the light, the diminution of heat, absence of smoke and objectionable by-products, and its economical advantages. As Dr. Welsbach remarks: "Lighting authorities cannot afford to disregard a burner which, with a consumption of 2·2 cubic feet, gives a light of 55 to 60 candle power, as against 12½ candle power given with five cubic feet. . . . It means nearly five times as much light with a saving of 2·8 cubic feet of gas per burner per hour. Taking 4,000 hours as the burning period of a street burner, that shows a yearly saving of over 11,000 cubic feet, which, at 2s. 6d. a thousand, would mean 27s. 6d. per burner per annum saved by the adoption of the system, and, at the same time, five times as much light as before." It is stated on behalf of the system that its expense is something like one-fifteenth that of electric light. The initial installation is, of course, far less than that of electric light; so that where economy is matter for consideration it is certain to be warmly welcomed. Every detail concerning the new burner can be had from the Welsbach Incandescent Gas Light Company (Limited), York Street, Westminster.

